# Prevalence of and Factors Associated with Hypertension in Children and Adolescents as Observed by German Pediatricians—A Case–Control Study

**DOI:** 10.3390/children12030348

**Published:** 2025-03-11

**Authors:** Jacob Christian Moll, Jens Bohlken, Karel Kostev

**Affiliations:** 1 Department of Pediatrics, Clinic Westbrandenburg, 14467 Potsdam, Germany; 2 Faculty of Medicine, Institute of Social Medicine, Occupational Health and Public Health, University of Leipzig, 04103 Leipzig, Germany; 3 University Hospital, Philipps-University Marburg, 35043 Marburg, Germany; 4 Epidemiology, IQVIA, 60549 Frankfurt am Main, Germany

**Keywords:** children, adolescents, hypertension, prevalence, incidence, risk factors

## Abstract

**Background:** Blood pressure elevation in children is an important health concern. The extent to which hypertension is diagnosed in German pediatric practices is not yet known. The aim of this study is, therefore, to examine the prevalence of hypertension diagnosis in children and adolescents treated in pediatric practices, as well as the factors associated with hypertension in this population. **Methods:** This retrospective case–control study used electronic medical records from 258 primary care pediatricians in Germany and included children and adolescents aged 0–17 years with an initial documented diagnosis of primary hypertension between January 2005 and December 2023. Hypertension patients were matched 1:5 with non-hypertension patients by age and sex. Conditional multivariable logistic regression models were used to estimate the association of chronic diseases and therapies with a risk of hypertension. **Results:** After 1:5 matching, the present study included 7482 children and adolescents with hypertension, and 37,410 controls without hypertension. The average prevalence of hypertension was 0.12% and the incidence was 1.24 cases per 1000 person-years, both increasing with age. In the multivariable regression analysis, a significant positive association was observed between hypertension and ten disorders including obesity (odds ratio, OR: 6.91; 95% confidence intervals, CI: 6.28–7.60), type 1 diabetes mellitus (OR: 2.85; 95% CI: 2.13–3.82), dyslipidemia (OR: 1.99; 95% CI: 1.46–2.72), chronic bronchitis (OR: 1.63; 95% CI: 1.39–1.90), hypothyroidism (OR: 1.62; 95% CI: 1.30–2.02), migraine (OR: 1.52; 95% CI: 1.17–1.98), ADHD (OR: 1.45; 95% CI: 1.28–1.65), scoliosis (OR: 1.40; 95% CI: 1.13–1.73), chronic rhinitis (OR: 1.31; 95% CI: 1.14–1.50), and reaction to severe stress and adjustment disorders (OR: 1.31; 95% CI: 1.04–1.65). Furthermore, paracetamol prescription was positively associated with hypertension risk (OR: 1.68; 95% CI: 1.41–2.00). **Conclusions:** The significant associations between hypertension and chronic disorders, particularly obesity, underscore the need for early prevention strategies. Prospective studies are needed to confirm these associations. Similarly, pathophysiological and mechanistic explanations for the associations identified need to be explored and verified in properly designed studies.

## 1. Introduction

Blood pressure elevation in children is an important health concern, as it translates to blood pressure elevation in adults, and can therefore have significant implications for cardiovascular health later in life [1]. European guidelines define elevated blood pressure in children under 16 years of age as a blood pressure level ≥95th percentile for sex, age, and height measured on at least three separate occasions. For adolescents aged 16 or older, the definition of hypertension is based on the absolute cut-off used for adults (≥140/90 mmHg) [2]. Finally, every hypertension evaluation includes a thorough anamnesis (family history, evidence of secondary hypertension, sleep history, information about general risk factors and medications), a physical examination, and basic diagnostics [2].

The International Statistical Classification of Diseases and Related Health Problems 10th Revision (ICD-10) includes two codes for hypertension: I10 for primary hypertension and I15 for secondary hypertension [3]. Usually, primary hypertension refers to high blood pressure in patients without any other medical condition or therapy which can potentially cause high blood pressure. Secondary hypertension may occur in connection with renal, endocrine, and other disorders [4].

The prevalence of elevated blood pressure in children in Germany was shown to be 10.4–15.6% from 2014 to 2017 [5], while a global meta-analysis has found the pooled prevalence of hypertension in children to be 4% [6]. It has already been demonstrated that blood pressure elevation is underdiagnosed in the US, where only 2.2–7.3% of children with blood pressure elevation were diagnosed with the condition [7]. High blood pressure in children and adolescents is often asymptomatic and can easily be overlooked for long periods [8].

Hypertension prevention in children and adolescents is usually discussed in the context of obesity treatment, preventive health, nutrition, and social counseling because these are believed to be connected to the most important risk factors for the development of the condition [9,10,11].

Previous studies have identified obesity, diabetes mellitus, primary hyperaldosteronism, renal disorders such as renal artery stenosis, coarctation of the aorta, systemic arteritis, and neurological disorders as well-known conditions that are associated with hypertension in children [12,13,14,15]. Furthermore, some medications can cause hypertension in susceptible individuals; these include nonsteroidal anti-inflammatory drugs, drugs used to treat attention-deficit/hyperactivity disorder (amphetamine, methylphenidate, dextroamphetamine), antidepressants, systemic corticosteroids, and others [12].

The frequency with which hypertension is diagnosed in German pediatric practices remains unclear. In this country, pediatricians oversee medical care for children up to the age of twelve. Beyond that, general practitioners (GPs) commonly take over their care, though over 30% of adolescents aged 13 to 17 continue to be seen by pediatricians. The country boasts some of the shortest physician wait times in Europe, and same-day visits to pediatric clinics are common where deemed medically necessary [16]. In view of the above, the aim of this study was to examine the prevalence of hypertension diagnosis in children and adolescents in pediatric practices, as well as the factors associated with hypertension in this population.

## 2. Methods

### 2.1. Database

This study used anonymized electronic medical records from the IQVIA^TM^ Disease Analyzer database including baseline demographic variables (age, sex), diagnoses, and prescriptions. The database is populated directly from computer systems used by approximately 3000 office-based general practitioners and specialists in Germany, including 258 primary care pediatricians [17].

The Disease Analyzer database is based on summary statistics for all physicians in Germany, which IQVIA uses to select physicians based on the following strata: specialist group, German federal state, community size category, and age of physician. The database has already been demonstrated to be representative of the country [17], and it has already been used in several studies focusing on the pediatric population [18,19,20].

### 2.2. Study Population

**Figure 1:**
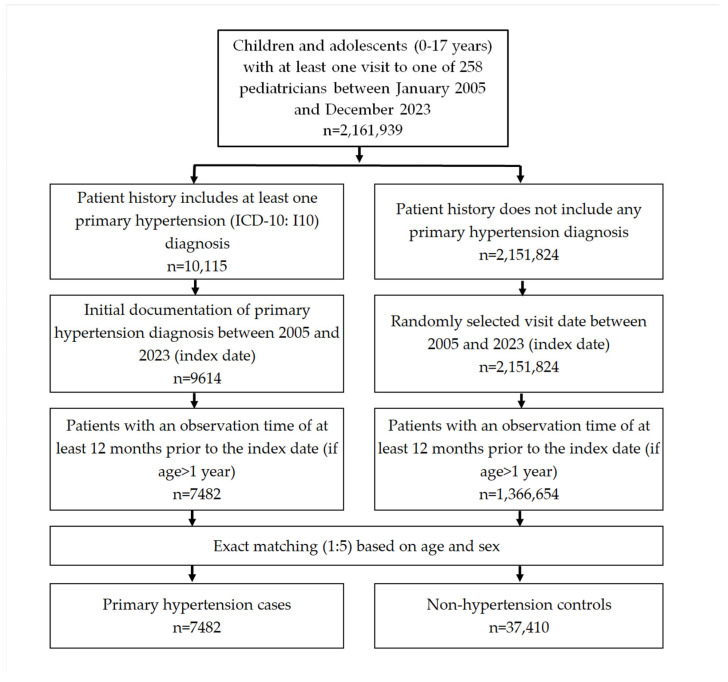
Selection of study patients.

This retrospective case–control study included children and adolescents aged 0–17 years with an initial diagnosis of primary hypertension (ICD-10: I10) documented by 1 of 258 pediatricians between January 2005 and December 2023. Children who were at least two years old had to have an observation time of at least 12 months prior to the first hypertension diagnosis so that all further diagnoses during this period could be analyzed. Hypertension patients were matched 1:5 with non-hypertension patients by age and sex. The index date was the date of hypertension diagnosis for participants with hypertension and that of a randomly selected pediatrician visit between January 2005 and December 2023 for those without hypertension (Figure 1). Secondary hypertension was rarely documented (*n* = 138) in children and adolescents, and therefore not included in further analysis.

### 2.3. Study Outcomes and Covariates

The study outcomes were:

(1) The prevalence and incidence of hypertension in children and adolescents overall and by age group (≤5, 6–12, 13–17 years) and sex;
(2) Disorders documented within 365 days prior to the index date that were significantly associated with the presence of an ICD-10 diagnosis of primary hypertension. All chronic diagnoses which occurred in at least 1.0% of the study population were considered; these included hypothyroidism (ICD-10: E03), type 1 diabetes mellitus (ICD-10: E10), obesity (ICD-10: E66), dyslipidemia (ICD-10: E78), reaction to severe stress and adjustment disorders (ICD-10: F43), somatoform disorders (ICD-10: F45), attention-deficit hyperactivity disorders (ICD-10: F90), epilepsy (ICD-10: G40), migraine (ICD-10: G43), vasomotor and allergic rhinitis (ICD-10: J30), chronic rhinitis (ICD-10: J31), chronic sinusitis (ICD-10: J32), chronic bronchitis (ICD10: J42), asthma (ICD-10: J45), atopic dermatitis (ICD-10: L20), and scoliosis (M41). Primary hyperaldosteronism (*n* = 0) and renal artery stenosis (*n* = 4) are extremely rare conditions, and were therefore not included in the analyses;
(3) Drugs prescribed within one month prior to the index date that were significantly associated with risk of hypertension. All active ingredients prescribed in at least 1.0% of the study population were considered. These therapies included ibuprofen (anatomical therapeutic chemical code (ATC): M01AE01), paracetamol (ATC: N02BE01), acetylcysteine (ATC: R05CB01), ambroxol (ATC: R05CB06), amoxicillin (ATC: J01CA04), xylometazoline (ATC: R01AB06), salbutamol (ATC: R03AC02), dimenhydrinate (ATC: R06AA02), and methylphenidate (methylphenidate).

### 2.4. Statistical Analysis

The prevalence of hypertension was calculated as the number of children with hypertension diagnosis divided by the number with documented visits to pediatricians. Hypertension incidence was calculated as the number of children and adolescents with initial hypertension diagnosis per 1000 person years based on the total follow-up time for all children and adolescents in the database.

Conditional multivariable logistic regression models were used to estimate chronic diseases and therapies associated with a risk of hypertension, including hypertension as an outcome variable and all disorders and drugs previously listed as independent variables. Regression analyses were performed for each age group. *p*-Value < 0.01 were considered statistically significant. Analyses were conducted using SAS version 9.4 (SAS Institute, Cary, CA, USA).

## 3. Results

### 3.1. Prevalence and Incidence of Hypertension

**Figure 2:**
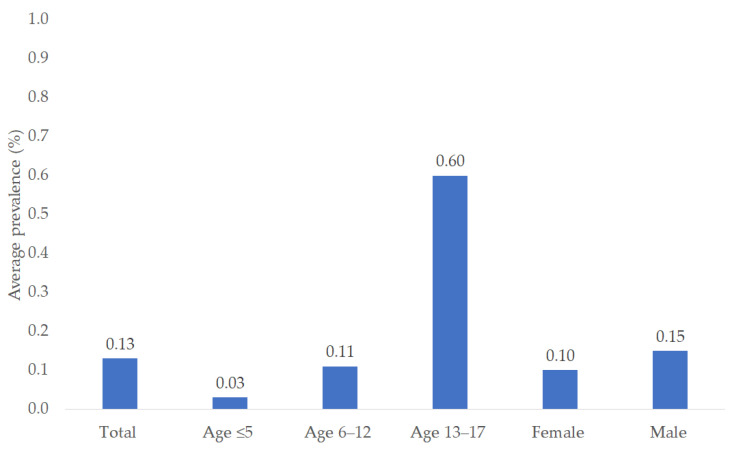
Average prevalence (2005–2023) of hypertension diagnosis in children and adolescents followed by 258 pediatricians in Germany.

Of 2,161,939 children and adolescents treated by 258 pediatricians, 10,115 had a diagnosis of hypertension documented between 2005 and 2023. The average prevalence per year was 0.12%, which was similar to the figures for 2005 (0.12%), 2014 (0.13%), and 2023 (0.12%). The average yearly prevalence was 0.03% in children aged ≤5 years, 0.11% in children aged 6–12, and 0.60% in adolescents (13–17 years). The yearly prevalence was slightly higher in males (0.15%) than in females (0.10%) (Figure 2).

**Figure 3:**
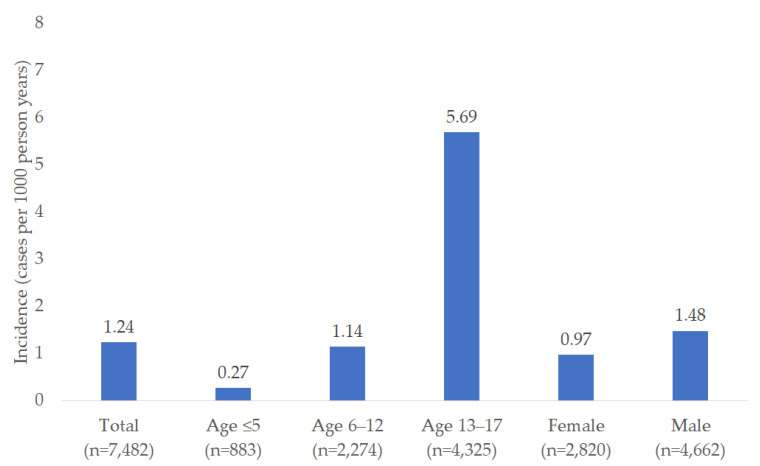
Incidence (cases per 1000 person-years) of hypertension diagnosis in children and adolescents followed by 258 pediatricians in Germany.

The incidence of hypertension diagnosis per 1000 person-years was 1.24 overall, increasing from 0.27 in children aged ≤5 years to 5.69 in the age group 13–17 years. The incidence was slightly higher in males (1.48 cases per 1000 person-years) than in females (0.97 cases per 1000 person-years) (Figure 3).

### 3.2. Basic Characteristics of the Study Sample

After 1:5 matching, the present study included 7482 children and adolescents with hypertension and 37,410 controls without hypertension. The basic characteristics of the study patients are listed in Table 1. The mean age [SD] was 11.8 [SD: 4.5] years; 37.7% were female.

Most patients were adolescents (57.8%), 30.4% were children aged 6–12 years, and 11.8% were aged ≤5 years.

### 3.3. Association of Pre-Defined Disorders with Arterial Hypertension

**Figure 4:**
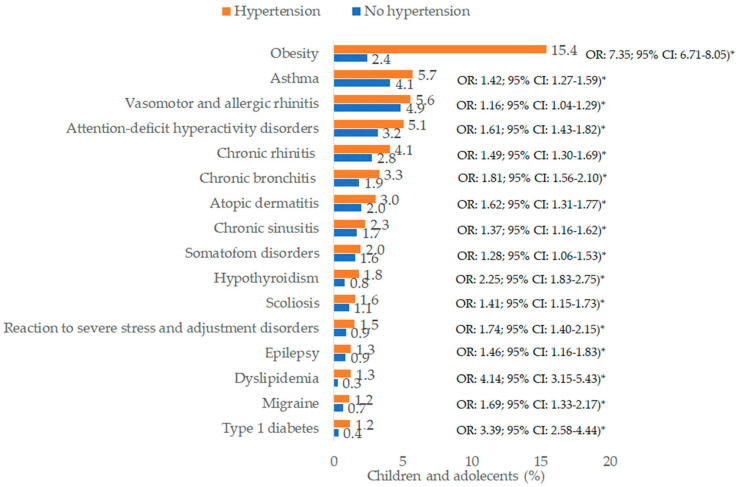
Prevalence of various disorders documented within 12 months prior to the index date in children with and without hypertension. * OR = odds ratio resulting from univariable logistic regression with hypertension as a dependent variable and each co-diagnosis as an independent variable; *p*-value marked with * are <0.01.

Figure 4 shows the prevalence of pre-defined chronic disorders and univariable odds ratios. The diagnosis with the highest prevalence was obesity (15.4% of hypertension cases and 2.4% of controls), followed by asthma (5.7% of hypertension cases and 4.1% of controls), vasomotor and allergic rhinitis (5.6% of hypertension cases and 4.9% of controls), ADHD (5.1% of hypertension cases and 3.2% of controls), and chronic rhinitis (4.1% of hypertension cases and 2.8% of controls).

**Figure 5:**
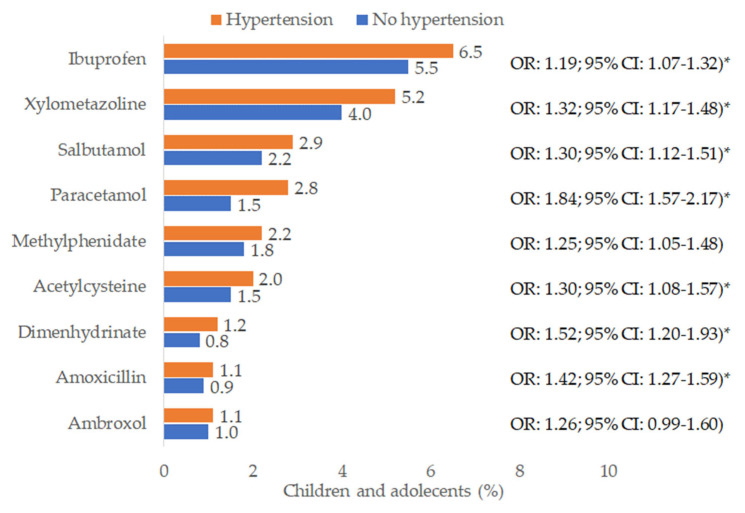
Prevalence of various therapies prescribed within three months prior to the index date in children with and without hypertension. * OR = odds ratio resulting from univariable logistic regression with hypertension as a dependent variable and each therapy as an independent variable; *p*-values marked with * are <0.01.

Figure 5 shows the prevalence of predefined therapies and univariable odds ratios. The therapy with the highest prevalence was ibuprofen (6.5% of hypertension cases and 5.5% of controls), followed by xylometazoline (5.2% of hypertension cases and 4.0% of controls), salbutamol (2.9% of hypertension cases and 2.2% of controls), and paracetamol (2.8% of hypertension cases and 1.6% of controls).

The adjusted odds ratios are shown in Table 2. In the multivariable regression analysis, a significant positive association was observed between hypertension and the following ten disorders: obesity (odds ratio, OR: 6.91; 95% confidence intervals, CI: 6.28–7.60), type 1 diabetes mellitus (T1DM) (OR: 2.85; 95% CI: 2.13–3.82), dyslipidemia (OR: 1.99; 95% CI: 1.46–2.72), chronic bronchitis (OR: 1.60; 95% CI: 1.36–1.87), hypothyroidism (OR: 1.62; 95% CI: 1.30–2.02), migraine (OR: 1.52; 95% CI: 1.17–1.98), ADHD (OR: 1.45; 95% CI: 1.28–1.65), scoliosis (OR: 1.38; 95% CI: 1.11–1.72), chronic rhinitis (OR: 1.28; 95% CI: 1.11–1.47), and reaction to severe stress and adjustment disorders (OR: 1.31; 95% CI: 1.04–1.65). However, although the associations were significant within the analyzed group as a whole, their strength varied by age group in some cases. Only two disorders were significantly associated with risk of hypertension in all three age groups (obesity and chronic bronchitis). The association was significant for T1DM, dyslipidemia, and ADHD in the age groups 6–12 and 13–17 years. Migraine was significantly associated with hypertension risk only in adolescents, while the association between hypertension risk and severe stress and adjustment disorders was significant only in the group of 6–12-year-olds. Scoliosis (OR: 12.79; 95% CI: 5.81–28.13), epilepsy, (OR: 7.13; 95% CI: 3.46–14.71), and hypothyroidism (OR: 7.45; 95% CI: 2.08–26.72) were strongly associated with hypertension in children aged ≤5 years.

## 4. Discussion

This study provides an analysis of pediatric hypertension diagnosis with respect to its prevalence, incidence, and associated comorbidities over nearly two decades (2005–2023) using data from over 2.1 million children and adolescents cared for by 258 pediatricians. Our study is the first in Germany to report the real-world prevalence of hypertension diagnosis in children and adolescents.

The average prevalence and yearly incidence of pediatric hypertension varied significantly by age, being lowest in children aged ≤5 years and highest in adolescents aged 13–17 years. These findings align with physiological changes in blood pressure during growth and development, as well as the impact of lifestyle factors such as diet, physical activity, and obesity, which tends to intensify during adolescence. However, the prevalence and incidence rates we observed were lower than those in published studies from other countries [5,6].

The slightly higher prevalence and incidence rates in males compared to females are consistent with the findings of previous studies that have identified sex-based differences in hypertension risk during childhood and adolescence [21,22]. Such differences are influenced by various factors, including body composition, hormonal differences, and cardiovascular health trajectories [23].

We found twelve conditions to be significantly associated with an increased risk of hypertension in children and adolescents in at least one age group. The strongest association was observed for obesity, with an odds ratio of 6.91. This robust relationship underscores the significance of obesity as a modifiable risk factor for hypertension, as already reported in previous studies [24,25,26].

Other comorbidities significantly associated with hypertension in the present study were type 1 diabetes mellitus (T1DM) (OR: 2.85) and dyslipidemia (OR: 1.99). These associations were observed in older children and adolescents in particular. Hypertension is considered a common complication in adolescents with T1DM [27,28,29]. Along with angiotensin I and II and inflammatory cytokines, persistent hyperglycemia plays a role in the development of hypertension in children with T1DM [30].

Dyslipidemia and hypertension are closely related conditions in children and adolescents, often co-occurring and sharing common risk factors such as obesity and poor diet [31]. Insulin resistance, which is frequently observed in obese children, serves as a common underlying factor connecting dyslipidemia and hypertension. It results in hyperinsulinemia, which is linked to both increased blood pressure and unfavorable lipid profiles [32].

The associations between chronic bronchitis and hypertension (OR: 1.60) and asthma and hypertension (OR: 2.11) may be linked to inflammation, as both conditions are characterized by ongoing inflammation of the airways, which can lead to systemic inflammation [33]. For instance, Zhang et al. examined the significant relationships between systemic inflammation markers and the prevalence of hypertension among children and adolescents aged 8 to 17 years in the United States, and found a significant association between such markers and hypertension risk [34]. This positive association between bronchitis and hypertension [35] and asthma and hypertension [36,37] has also been reported in previous studies.

The association between chronic rhinitis and hypertension (OR: 1.28) is a further interesting finding. Previous research has firmly established a link between rhinitis and obstructive sleep apnea (OSA), along with its key symptoms of snoring and daytime sleepiness due to the inherent nasal obstruction caused by rhinitis. In turn, OSA and snoring directly impact arterial blood pressure, also causing hypertension [38].

The association between hypothyroidism and hypertension (OR: 1.62 in the overall population and OR: 7.45 in children aged ≤5 years) observed in the present study is also noteworthy. Increased peripheral vascular resistance and reduced cardiac output have been proposed as potential reasons for the link between hypothyroidism and diastolic hypertension. Individuals with hypothyroidism experience significant changes in blood volume, triggering a volume-dependent blood pressure elevation mechanism which is associated with low plasma renin activity [39].

The relationship between hypertension and conditions such as ADHD (OR: 1.45), migraine (OR: 1.52), and reaction to severe stress (OR: 1.31) points to a potential link between hypertension and neuropsychological or stress-related disorders. Indeed, children with ADHD are commonly prescribed psychostimulants, which can cause a moderate rise in blood pressure [40]. We have not been able to pinpoint specific reasons for the epidemiological association between migraine and arterial hypertension identified in this database study. Some authors have hypothesized that individuals with migraine may have increased aortic stiffness and/or sub-clinical atherosclerosis [41,42].

Regarding the association between severe stress and hypertension, there are two possible explanations. First, psychological stress triggers a rise in blood pressure by activating the sympathetic nervous system when a person is experiencing stress, and blood pressure may remain elevated even after the stress subsides rather than quickly returning to pre-stress levels [43]. Second, mental stress elevates cortisol levels, leading to endothelial dysfunction and disrupted blood pressure regulation [44]. Although there is no evidence as yet of a similar effect in children, it can reasonably be assumed that this may be the case.

A further strong association (OR: 7.13) observed in our study was that between epilepsy and hypertension in children aged ≤5 years. Seizure activity can lead to an increase in blood pressure, likely due to its impact on different neuronal networks within the central autonomic nervous system [45].

The last strong association (OR: 12.79) was observed between scoliosis and hypertension in children aged ≤5 years. However, the possible pathophysiological link between scoliosis and arterial hypertension remains unclear. It could be hypothesized that children with scoliosis may frequently suffer from neurodevelopmental disorders, which in turn are often associated with sedentary behavior, obesity, and poor cardiovascular health. However, no such hypothesis has yet been verified.

Finally, the prescription of paracetamol was significantly associated with an increased risk of hypertension diagnosis in all age groups. As drugs such as paracetamol can be bought without prescription in Germany, it is difficult to discern whether this is a causal association or a confounding effect (children taking paracetamol often have a fever and/or pain, which may be accompanied by increased blood pressure, which could be documented as blood pressure elevation if they visit a pediatrician during this time). As yet, there is no pathophysiological explanation for such an association.

### 4.1. Clinical and Public Health Implications

Arterial hypertension is a significant concern in pediatrics, especially given its tendency to persist into adulthood and its long-term impact on cardiovascular health [46,47]. The increasing incidence of hypertension from 0.27 cases per 1000 person-years in children aged ≤5 years to 5.69 cases in adolescents underscores the need for management strategies tailored to different age groups. One critical finding of this study is the association between hypertension and various comorbid conditions, particularly obesity. This highlights the role of lifestyle factors in the development of hypertension and suggests that public health initiatives should focus on promoting healthy weight management among children and adolescents. The fact that obesity and chronic bronchitis were significantly associated with hypertension across all age groups indicates that these conditions should be prioritized in both clinical practice and public health strategies. In addition, both pediatricians and general practitioners should be trained to recognize the signs of hypertension early on and implement routine screening, especially in high-risk populations.

### 4.2. Strengths and Limitations

This study’s strengths include its large sample size, extended follow-up period, the large number of different variables considered, and its robust matching design. However, it also has several limitations that warrant consideration. First, the study relied on diagnoses recorded by pediatricians, which may under- or overestimate the true prevalence of arterial hypertension. In particular, the ICD-10 code for hypertension used by pediatricians may cause a bias in the present study. Second, the database contains ICD-10 codes for diagnoses, but no details on the diagnosis or screening processes. For example, no data are available regarding 24 h ambulatory blood pressure measurement. However, diagnoses documented by pediatricians may incorporate diagnoses made by other specialists, including cardiologists, nephrologists or hospital-based outpatient clinics where ambulatory blood pressure monitoring might have been performed. Third, the database used does not include information on family history of hypertension or other diseases. Fourth, no data were available on breastfeeding in infancy. Fifth, no information on lifestyle factors such as smoking and alcohol use in adolescents and nutrition as well as sedentary lifestyle was available in the database. Sixth, the present study focuses on primary hypertension. As secondary hypertension is rarely diagnosed, the number of patients in the database with this diagnosis does not allow for a detailed analysis of the factors associated with secondary hypertension. However, the results of this study indicate that a large number of children included in this study have diagnoses which should be coded as a secondary hypertension.

Finally, this study used a retrospective case–control design, which does not allow conclusions to be drawn regarding causal effects, but only statistical associations.

## 5. Conclusions

The significant associations between hypertension and a number of chronic disorders, particularly obesity, underscore the need for early prevention strategies. Prospective studies are needed to confirm these associations. Similarly, pathophysiological and mechanistic explanations for the associations identified need to be explored and verified in properly designed studies.

## Figures and Tables

**Table 1:** Age and sex characteristics of study patients after 1:5 matching.

Variable	Proportion Among Children and Adolescents with Hypertension (N, %)	Proportion Among Children and Adolescents Without Hypertension (N, %)	*p*-Value
N	7482	37,410	
Sex: female	2820 (37.7)	14,100 (37.7)	1.000
Age (mean, SD)	11.8 (4.5)	11.8 (4.5)	1.000
Age ≤5 years	882 (11.8)	4415 (11.8)	1.000
Age 6–12 years	2274 (30.4)	11,370 (30.4)	
Age 13–17 years	4325 (57.8)	21,625 (57.8)	

**Table 2:** Association between several disorders and arterial hypertension diagnosis in children and adolescents followed by pediatricians in Germany (multivariable logistic regression, only significant associations are displayed).

	Adjusted Odds Ratio for Hypertension (95% CI) *			
Diagnosis documented within 12 months prior to the index date and therapies prescribed within 3 months prior to the index date	Total population	Age ≤5 years	Age 6–12 years	Age 13–17 years
Obesity	6.91 (6.28–7.60)	9.17 (5.72–14.68)	8.75 (7.43–10.29)	5.96 (5.28–6.74)
Type 1 diabetes mellitus	2.85 (2.13–3.82)	–	3.24 (1.66–6.36)	2.76 (2.00–3.81)
Dyslipidemia	1.99 (1.46–2.72)	–	3.64 (1.94–6.81)	1.68 (1.16–2.42)
Chronic bronchitis	1.60 (1.36–1.87)	1.68 (1.26–2.24)	1.51 (1.12–2.03)	1.51 (1.17–1.96)
Hypothyroidism	1.62 (1.30–2.02)	7.45 (2.08–26.72)	3.18 (1.94–5.20)	–
Migraine	1.52 (1.17–1.98)	–	–	1.70 (1.27–2.27)
Attention-deficit hyperactivity disorders	1.45 (1.28–1.65)	–	1.54 (1.24–1.89)	1.41 (1.20–1.66)
Scoliosis	1.38 (1.11–1.72)	12.79 (5.81–28.13)	–	–
Chronic rhinitis	1.28 (1.11–1.47)	1.82 (1.41–2.36)	–	–
Epilepsy	–	7.13 (3.46–14.71)	–	–
Asthma	–	2.11 (1.35–3.30)	–	–
Reaction to severe stress and adjustment disorders	1.31 (1.04–1.65)	–	1.78 (1.18–2.68)	–
Paracetamol	1.68 (1.41–2.00)	1.63 (1.27–2.57)	1.71 (1.19–2.45)	1.61 (1.11–2.34)

* Multivariable conditional logistic regression adjusted for all diagnoses listed in the table; *p* < 0.01 is considered statistically significant.

## Data Availability

The data presented in this study are available on request from the corresponding author due to privacy reasons.
